# Metabolomic Analysis Reveals Contributions of Citric and Citramalic Acids to Rare Earth Bioleaching by a *Paecilomyces* Fungus

**DOI:** 10.3389/fmicb.2019.03008

**Published:** 2020-01-14

**Authors:** Vanessa L. Brisson, Wei-Qin Zhuang, Lisa Alvarez-Cohen

**Affiliations:** ^1^Department of Civil and Environmental Engineering, University of California, Berkeley, Berkeley, CA, United States; ^2^Biosciences and Biotechnology Division, Physical and Life Sciences Directorate, Lawrence Livermore National Laboratory, Livermore, CA, United States; ^3^Earth Sciences Division, Lawrence Berkeley National Laboratory, Berkeley, CA, United States

**Keywords:** bioleaching, rare earth elements, thorium, metabolomics, *Paecilomyces*, phosphate solubilizing

## Abstract

Conventional methods for extracting rare earth elements from monazite ore require high energy inputs and produce environmentally damaging waste streams. Bioleaching offers a potentially more environmentally friendly alternative extraction process. In order to better understand bioleaching mechanisms, we conducted an exo-metabolomic analysis of a previously isolated rare earth bioleaching fungus from the genus *Paecilomyces* (GenBank accession numbers KM874779 and KM 874781) to identify contributions of compounds exuded by this fungus to bioleaching activity. Exuded compounds were compared under two growth conditions: growth with monazite ore as the only phosphate source, and growth with a soluble phosphate source (K_2_HPO_4_) added. Overall metabolite profiling, in combination with glucose consumption and biomass accumulation data, reflected a lag in growth when this organism was grown with only monazite. We analyzed the relationships between metabolite concentrations, rare earth solubilization, and growth conditions, and identified several metabolites potentially associated with bioleaching. Further investigation using laboratory prepared solutions of 17 of these metabolites indicated statistically significant leaching contributions from both citric and citramalic acids. These contributions (16.4 and 15.0 mg/L total rare earths solubilized) accounted for a portion, but not all, of the leaching achieved with direct bioleaching (42 ± 15 mg/L final rare earth concentration). Additionally, citramalic acid released significantly less of the radioactive element thorium than did citric acid (0.25 ± 0.01 mg/L compared to 1.18 ± 0.01 mg/L), suggesting that citramalic acid may have preferable leaching properties for a monazite bioleaching process.

## Introduction

Rare earth elements (REEs) are critical for a variety of technologies (US DOE, 2011; Alonso et al., 2012). Monazite, a rare earth phosphate mineral, is one of the main ores used for commercial REE production (Gupta and Krishnamurthy, 1992; Rosenblum and Fleischer, 1995). However, conventional monazite extraction methods involve chemical leaching at high temperatures, and are energy intensive and environmentally damaging (Merritt, 1990; Gupta and Krishnamurthy, 1992; Alonso et al., 2012; Peelman et al., 2014). Additionally, the radioactive element thorium (Th) is also usually present along with REE-phosphates in monazite ore (Rosenblum and Fleischer, 1995). Conventional monazite extraction methods co-extract Th, which must be separated from REEs in downstream processing (Gupta and Krishnamurthy, 1992).

Bioleaching offers a possible alternative to conventional REE extraction, potentially resulting in a more environmentally sustainable extraction process (Brisson et al., 2016; Fathollahzadeh et al., 2018a). Several previous studies have investigated the potential for bioleaching of REEs from monazite by phosphate solubilizing microorganisms (PSMs), including both fungi (genera: *Aspergillus*, *Paecilomyces*, and *Penicillium*) and bacteria (genera: *Acidithiobacillus*, *Bacillus*, *Burkholderia*, *Enterobacter*, *Klebsiella*, *Microbacterium*, *Pantoea*, *Pseudomonas*, and *Streptomyces*) (Brisson et al., 2016; Corbett et al., 2017, 2018; Fathollahzadeh et al., 2018b, c, 2019). Monazite bioleaching by some fungi has also been shown to preferentially release REEs into solution over Th (Brisson et al., 2016). Several recent studies have investigated bioleaching REEs from substrates other than monazite. This includes leaching REEs from bastnaesite, another important REE bearing ore (Zhang et al., 2018), and bioleaching to recover REEs from waste products including spent catalysts, rare earth magnets, and coal fly ash (Auerbach et al., 2019; Jin et al., 2019; Park and Liang, 2019).

Phosphate solubilizing microorganisms are microorganisms that have the ability to solubilize phosphate ions from otherwise insoluble phosphate compounds and minerals (Rodríguez and Fraga, 1999). Current understanding of the mechanisms of phosphate solubilization by PSMs indicates that three main contributing factors are acidification of the medium, exchange reactions, and the formation of complexes between organic acids or other chelating molecules produced by the PSMs and cations associated with phosphate in the mineral and released during solubilization (Bolan et al., 1994; Rodríguez and Fraga, 1999; Nautiyal et al., 2000; Gyaneshwar et al., 2002; Arcand and Schneider, 2006; Scervino et al., 2011). Previous work on monazite bioleaching indicated that although both acidification and complexation with citric acid were capable of contributing to monazite leaching, these contributions did not account for the levels of leaching seen during bioleaching or when leaching with spent bioleaching medium (Brisson et al., 2016). This indicates that other compounds released into solution, but not identified by the methods used in that study, contribute to the bioleaching process.

Mass spectrometry based metabolomics technologies provide the opportunity to accurately detect a large number of different organic molecules and compare relative concentrations across different conditions, providing insight into biological processes. Metabolomic analyses applied to exuded metabolites are sometimes referred to as exometabolomics or metabolic footprinting (Kell et al., 2005). Metabolic footprinting has been applied to investigate other eukaryotic microbial processes including wine production and microalgae growth in bioreactors (Howell et al., 2006; Sue et al., 2011; Richter et al., 2013). Recent metabolomic studies of fungi have investigated a variety of important processes including the production of mycotoxins, fungal degradation of biomass waste, and metabolic changes and metabolite exchange in fungal-bacterial mutualistic interactions (Karpe et al., 2015, 2018; Shi et al., 2017; Uehling et al., 2019). One recent study did a comparative genomic and exometabolomic analysis of 27 members of *Aspergillus* section *Nigri*, a group of fungi with important roles in biotechnology, the environment, and human health (Vesth et al., 2018).

A metabolomics approach could potentially enable better understanding of the bioleaching process by identifying a larger array of small molecules released during bioleaching that might be associated with bioleaching effectiveness. In this study, we analyzed metabolites exuded into the growth medium during monazite bioleaching under two different growth conditions: growth with monazite as the only phosphate source (using soluble phosphate limitation to force monazite solubilization) and growth with the addition of a soluble phosphate source (relieving the phosphate limitation stress). This analysis had two parallel goals. One was to examine the effects of phosphate availability on growth and metabolic processes of a bioleaching microorganism, and the second was to identify metabolites exuded into solution that may contribute to monazite solubilization. Once those metabolites of interest were identified, the ability of laboratory prepared solutions of those metabolites to leach monazite under abiotic conditions was studied to further investigate individual metabolites’ contributions to bioleaching.

## Materials and Methods

### Organism and Bioleaching Growth Conditions

Bioleaching experiments were conducted with a monazite bioleaching fungal isolate designated WE3-F (GenBank accession numbers KM874779 and KM874781), whose isolation and identification as a *Paecilomyces* species were described previously (Brisson et al., 2016). This organism was selected for further study based on its consistent bioleaching performance in that study.

Growth conditions were based on those described previously (Brisson et al., 2016) with some modifications. Briefly, bioleaching was conducted in 250 mL glass Erlenmeyer flasks, each containing 0.5 g ground monazite sand (City Chemical LLC, West Haven, CT, United States) (finer than 200 mesh) and 50 mL modified ammonium salts medium (AMS medium) (Parales et al., 1994). AMS medium contained 1.0 g/L MgSO_4_⋅7H_2_O, 0.2 g/L KCl, 0.66 g/L (NH_4_)_2_SO_4_, 1.0 mL/L 1000× trace elements stock solution, and 1.0 mL/L stock A. The 1000× trace elements stock solution contained 0.5 g/L FeSO_4_⋅7H_2_O, 0.4 g/L ZnSO_4_⋅7H_2_O, 0.02 g/L MnSO_4_⋅H_2_O, 0.015 g/L H_3_BO_3_, 0.01 g/L NiCl_2_⋅6H_2_O, 0.25 g/L EDTA, 0.05 g/L CoCl_2_⋅6H_2_O, and 0.005 g/L CuCl_2_⋅2H_2_O. Stock A contained 5 g/L FeNaEDTA and 2 g/L NaMoO_4_⋅2H_2_O. 10 g/L glucose was added as a carbon and energy source and air in the headspace served as oxygen source. Each flask was inoculated with 1 mL of spore suspension containing approximately 10^7^ CFU and sealed with a foam stopper. As in previous bioleaching studies with this organism (Brisson et al., 2016), flasks were stirred continuously at 250 rpm to ensure well mixed conditions, and incubated at 28°C for the duration of the bioleaching experiment.

Two different growth conditions were compared to study the effects of a soluble phosphate source: growth with monazite only and growth with K_2_HPO_4_ and monazite. For each K_2_HPO_4_ and monazite condition flask, 0.4 g/L K_2_HPO_4_ was added.

### Quantification of REEs, Th, Phosphate, Glucose, pH, and Biomass

Rare earth element, Th, phosphate, glucose, pH, and biomass were quantified by previously described analytical methods (Brisson et al., 2016). Briefly, REE and Th concentrations were measured using an Agilent Technologies 7700 series ICP-MS. Phosphate concentration was measured using the BioVision Phosphate Colorimetric Assay Kit. Glucose was measured by HPLC on a Waters 2695 HPLC system with a BioRad Aminex HPX-87H carbohydrate/organic acids analysis column and a Waters 2414 refractive index detector. pH was measured using a Hanna Instruments HI 2210 pH meter. Biomass was measured as total volatile solids of filter-collected samples by drying at 105°C and subsequent ashing at 550°C as described previously (Brisson et al., 2016) based on United States Environmental Protection Agency Method 1684 (US EPA, 2001). REE, Th, phosphate, glucose, and pH measurements were taken for six biological replicates for each time point (0, 2, 4, and 6 days after inoculation), while biomass measurements were taken for three biological replicates at time points 2, 4, and 6 days.

### Metabolomic Analysis

Samples of bioleaching supernatant were collected, filtered through 0.2 μm syringe filters to remove cells, and immediately frozen and stored at −80°C. Six replicate samples were collected at each time point (0, 2, 4, and 6 days after inoculation). The *t* = 0 days samples served as a media controls, since they consisted of medium that had not undergone incubation with fungi. Subsequent samples represent metabolite production (and consumption) during the bioleaching process. Metabolomic analysis was performed by the West Coast Metabolomics Center at the University of California, Davis.

Details of the protocols used for metabolite extraction, derivatization, gas chromatography-mass spectrometry (GC-MS) data acquisition, and data processing have been described in detail previously (Fiehn et al., 2008; Fiehn, 2016). Briefly, the samples were extracted with a 3:3:2 solution of acetonitrile, isopropanol, and water. Extracted samples were derivatized via silylation derivatization with N-Methyl-N-(trimethylsilyl)trifluoroacetamide (MSTFA). Derivatized samples were analyzed on an Agilent 6890 GC coupled with a Pegasus TOF MS. The GC column used was an Rtx-5Sil GC column (30 cm long, 0.25 mm internal diameter). Compound identifications were made based on their retention index and comparisons of their mass spectra to the BinBase metabolomics database using the BinBase algorithm (Skogerson et al., 2011).

Hierarchical clustering (Müllner, 2011) of metabolites based on concentration profiles was performed in Python using the SciPy cluster module. Signal intensity data for each metabolite were first centered by subtracting the mean signal intensity for that metabolite, and normalized by dividing by the standard deviation. Hierarchical clustering was performed using the “complete” method, also called the farthest point algorithm, with Euclidian distances (Müllner, 2011).

### Identification of Metabolites of Potential Bioleaching Importance

Metabolites that were potentially relevant to bioleaching performance were identified by three methods. The first method identified metabolites that were released at higher concentrations under the monazite-only condition than under the K_2_HPO_4_ plus monazite condition. Signal intensities for each metabolite were compared between the two conditions using a two-tailed *T*-test for independent samples. *p*-values were corrected for multiple comparisons using the Benjamini/Hochberg correction for false discovery rate for independent samples (Benjamini and Hochberg, 1995). For this analysis only, all metabolites for which the *p*-values were marginally significant (*p* < 0.1) were selected for further study. This less stringent *p*-value criterion was used at this intermediate stage in order to identify a large number of metabolites for the final set of experiments. This analysis was performed independently for time points 2, 4, and 6 days.

The second approach to selecting metabolites of interest was to identify correlations between metabolite concentration (signal intensity) and REE concentration over all time points. This analysis was performed on data from the monazite-only condition, using measurements of metabolite concentrations and REE concentrations at each time point. A least squares linear regression was performed to identify correlations. *p*-values were corrected for multiple comparisons using the Šidák correction (Šidák, 1967). Metabolites for which the linear regression had a positive slope and a significant corrected *p*-value (*p* > 0.05) were selected for further study.

The final approach was to select metabolites with the highest signal intensities. Metabolites for which the average signal intensity was greater than 10^5^ for any condition and time point were selected for further study.

### Abiotic Leaching With Identified Metabolites

Abiotic leaching conditions were conducted as previously described (Brisson et al., 2016), with some modifications. Leaching was conducted in 50 mL flat bottomed polypropylene tubes, each containing 0.1 g ground monazite sand (200 mesh). 10 mL leaching solution was added to autoclaved tubes and stirred for 48 h at 250 rpm at room temperature (25–28°C). All leaching solutions were tested in triplicate.

Leaching solutions contained selected metabolites at a concentration of 10 mM, with the exception of stearic acid. This concentration was selected based on observed concentrations of organic acids identified in our previous study of monazite bioleaching (Brisson et al., 2016). Stearic acid, which has an extremely low solubility in water (0.003 g/L or 0.01 mM at 20°C) (Anneken et al., 2000), was dissolved in water for 20 min with vortexing and filtered to remove undissolved particles. Additionally, a combined leaching solution containing all selected metabolites, each at a concentration of 10 mM (except for stearic acid), was also tested. All leaching solutions were adjusted to pH 2.5 by the addition of HCl in order to mimic the pH observed during bioleaching and to eliminate the effects of variations in pH observed previously (Brisson et al., 2016). By making this adjustment, contributions of individual metabolites could be quantified based on their ability to increase leaching beyond what was expected for that pH level. Leaching solutions were filter sterilized through 0.2 μm syringe filters prior to leaching experiments.

Statistical significance of leaching effectiveness was determined using a two-tailed *T*-test for independent samples to compare REEs released by each leaching solution to a control solution of HCl at a pH of 2.5. *p*-values were corrected for multiple comparisons using the Šidák correction (Šidák, 1967).

## Results

### Bioleaching Performance

**FIGURE 1:**
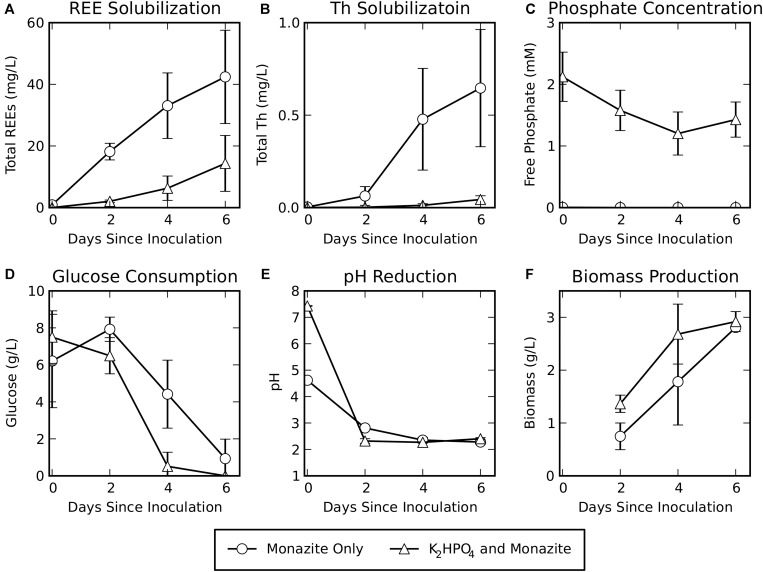
Bioleaching of monazite in the absence or presence of soluble phosphate (K2HPO4). Shown are **(A)** REE concentrations, **(B)** Th concentrations, **(C)** phosphate concentrations, **(D)** glucose concentrations, **(E)** pH, and **(F)** biomass measured as volatile solids. REE, phosphate, glucose, and pH data are for six biological replicates. Biomass data are for three biological replicates. Error bars indicate standard deviations around the mean.

Rare earth element solubilization was greater for the monazite-only condition, when a soluble phosphate source was not provided, reaching concentrations of 42 ± 15 mg/L total REEs after 6 days of leaching (Figure 1A). However, some solubilization of REEs did occur in the cultures provided with K_2_HPO_4_, reaching concentrations of 14 ± 9 mg/L after 6 days of bioleaching. Although release of the radioactive element Th was low for both conditions, it was consistently greater for the monazite-only condition (0.6 ± 0.3 mg/L) than for K_2_HPO_4_ plus monazite (0.04 ± 0.02 mg/L) (Figure 1B).

Free phosphate concentrations (Figure 1C) remained very low (maximum observed concentration in a single sample: 0.005 mM) when monazite was the only phosphate source. When K_2_HPO_4_ was added to the medium, phosphate levels decreased from their initial concentration but remained high throughout the experiment (minimum observed concentration in a single sample: 0.68 mM). This indicates that the concentration of K_2_HPO_4_ provided was sufficient to avoid phosphate limiting conditions during bioleaching for this growth condition.

Glucose consumption (Figure 1D) for the monazite-only growth condition lagged behind glucose consumption when K_2_HPO_4_ was provided. Similarly, the pH was reduced at a faster rate when soluble phosphate was provided (Figure 1E), resulting in a slightly lower pH for this condition on day two of bioleaching despite the higher initial pH of the medium with added K_2_HPO_4_. However, by the fourth day, both conditions had achieved a similar pH. Biomass production (Figure 1F) for the monazite-only condition also lagged behind growth with K_2_HPO_4_ plus monazite. By the sixth day, however, biomass accumulation was comparable under both growth conditions (2.8 ± 0.03 g/L for monazite-only and 2.9 ± 0.2 g/L for K_2_HPO_4_ with monazite).

### Overall Metabolomic Profile

**FIGURE 2:**
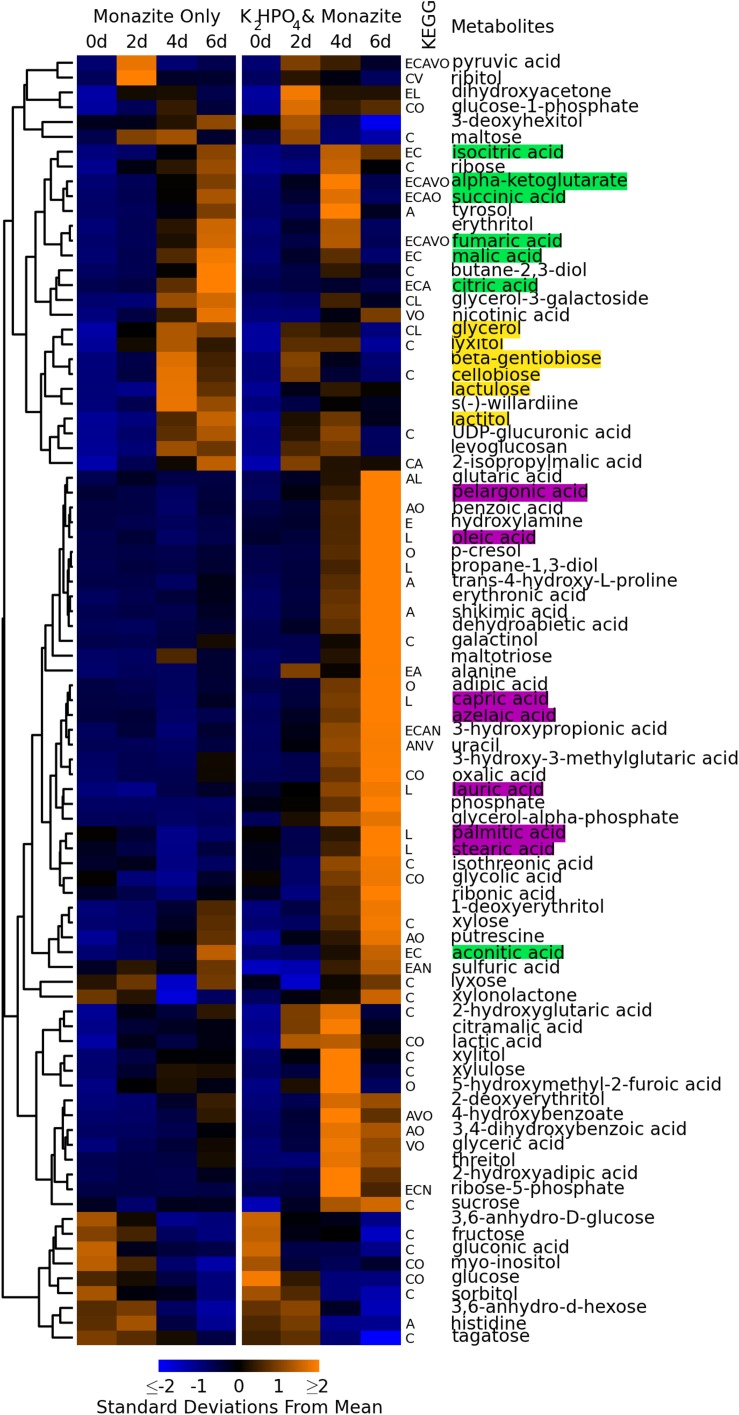
Concentration profiles of identified metabolites. Heatmap shows average levels of identified metabolites detected during monazite bioleaching for each growth condition and time point. Columns represent different conditions and time points. Rows represent different metabolites. Metabolites are ordered based on hierarchical clustering, with the clustering dendrogram displayed to the left of the heatmap and metabolite names to the right. Heatmap colors indicate standard deviations below (blue) and above (yellow) the overall mean level for each metabolite. Letters between heatmap and metabolite names indicate KEGG pathways involving each metabolite. E = energy metabolism, C = carbohydrate metabolism, A = amino acid metabolism, L = lipid metabolism, N = nucleic acid metabolism, V = metabolism of cofactors and vitamins, O = other KEGG pathways. Metabolites highlighted in green are part of the TCA cycle. Metabolites highlighted in yellow are disaccharides and alcohols detected early in the growth cycle. Metabolites highlighted in purple are long chain (>8 carbon) fatty acids.

Metabolomic analyses of the fungal supernatant from the two conditions detected 210 metabolites. Of these 87 could be identified as known compounds. The remaining 123 were identified only with BinBase ID numbers based on their characteristic mass spectra, referring to the BinBase metabolomics database (Skogerson et al., 2011). Details of the compound identifications and mass spectra are given in Supplementary Table 1, and peak heights for all identified compounds in all samples are given in Supplementary Table 2. Concentration profiles of all metabolites identified by chemical name are summarized in Figure 2 for all time points and conditions. Concentration profiles for all 210 metabolites are summarized in Supplementary Figure 1.

Nine identified compounds were detected at significant levels in the medium control (*t* = 0 days) samples (Figure 2, bottom nine rows). These included glucose, which was added to the medium as a carbon source. Because these were present in the medium, and did not increase with bioleaching, they were not considered to be fungal metabolites.

The concentration profiles of fungal metabolites were consistent with the lag in growth when monazite was the only phosphate source, observed above in glucose consumption, pH reduction, and biomass growth (see section “Bioleaching Performance,” Figures 1D–F). Early exuded metabolites (detected at day two for growth with K_2_HPO_4_ and day for monazite only) included several disaccharides (cellobiose, lactulose, and β-gentiobiose) and alcohols (lactitol, lyxitol, and glycerol) (Figure 2, yellow highlights).

The lag in growth for the monazite only condition was paralleled in the concentration time profiles for most metabolites involved in the tricarboxylic acid cycle (TCA cycle) (Figure 2, green highlights), including citric, isocitric, α-ketoglutaric, succinic, fumaric, and malic acids. The concentrations of these TCA cycle components peaked on day four when K_2_HPO_4_ was added and on day six when monazite was the only phosphate source. Aconitic acid, also in the TCA cycle, had a different concentration profile, peaking on day six for both growth conditions, while oxaloacetic acid was not detected.

A large group of metabolites (Figure 2, middle rows), were present at the highest levels on day six when K_2_HPO_4_ was provided, and remained at much lower levels when monazite was the only phosphate source. These included several long chain (more than eight carbons) fatty acids including azelaic, capric, lauric, oleic, palmitic, pelargonic, and stearic acids (Figure 2, purple highlights).

### Identification of Metabolites of Potential Bioleaching Importance

**FIGURE 3:**
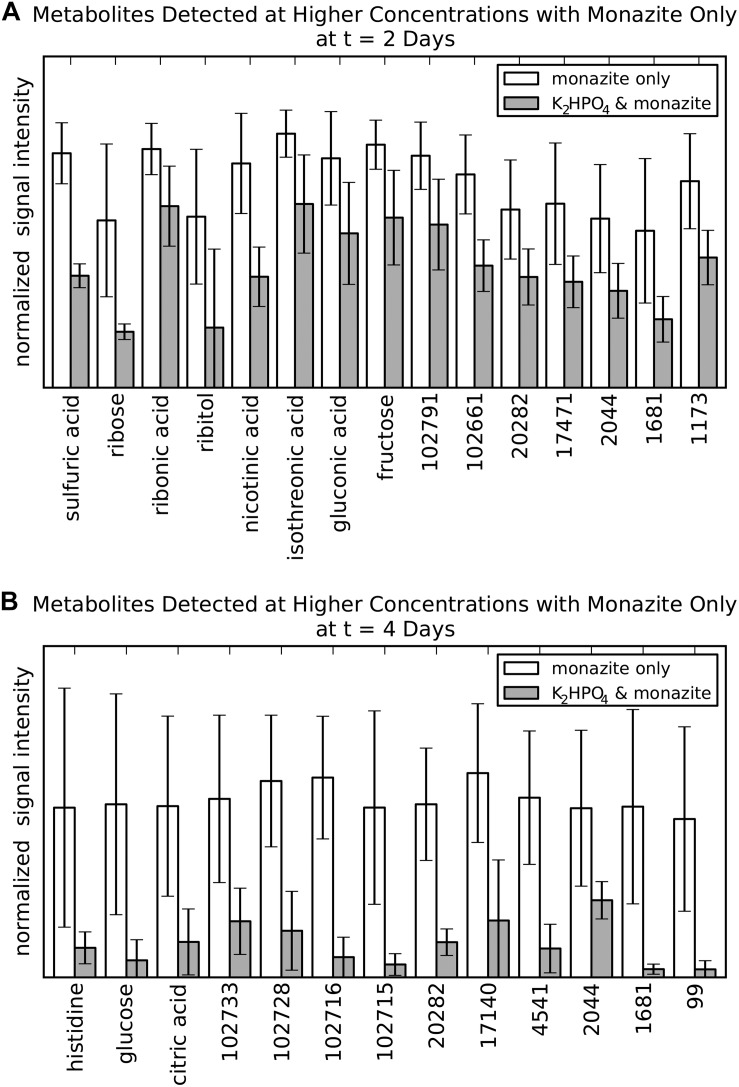
Metabolites of potential bioleaching importance identified by occurrence at higher concentrations in the monazite-only condition compared to K_2_HPO_4_ plus monazite. **(A)** Metabolites identified at 2 days. **(B)** Metabolites identified for at 4 days. Signal intensities are normalized as the fraction of the maximum observed signal intensity for each metabolite at that time point. Height and error bars indicate mean and standard deviation for six biological replicates. Metabolites selected based on a marginally significant Benjamini/Hochberg adjusted *p*-value < 0.1. This less stringent *p*-value criterion was used at this intermediate stage only, in order to identify a large number of metabolites for the abiotic leaching experiments.

**FIGURE 4:**
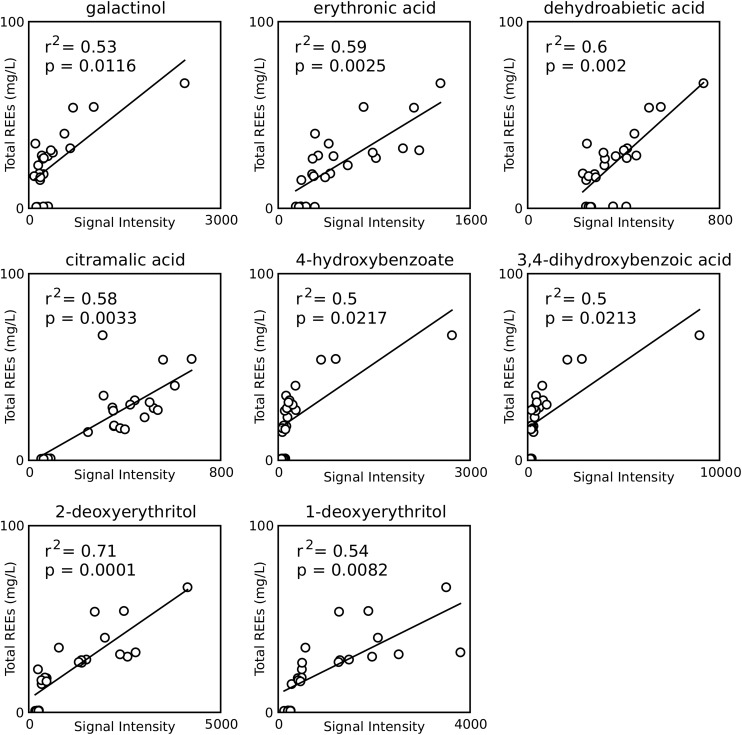
Correlations between metabolite signal intensities and REE concentrations for all samples from the monazite only condition. Only compounds found to have a significant positive correlation and having identification beyond BinBase ID numbers are shown. *p*-values are Šidák adjusted.

Metabolites of potential bioleaching importance were identified with three separate approaches: higher concentrations when soluble phosphate was not available (Figure 3), correlation with REE concentrations (Figure 4), and high signal intensity. Of those that could be identified by chemical name, a subset were selected for further study (Table 1).

**TABLE 1:** Metabolites of potential bioleaching importance that could be identified by chemical name.

		**Selected for**
**Identification method**	**Metabolite**	**abiotic leaching**
direct comparison day 2	sulfuric acid ^†^	
	Ribose	X
	ribonic acid ^‡^	
	Ribitol	X
	nicotinic acid	X
	isothreonic acid	X
	gluconic acid	X
	fructose ^§^	
direct comparison day 4	Histidine	X
	glucose ^¶^	
	citric acid	X
correlation with REE concentration	Galactinol	X
	erythronic acid ^‡^	
	dehydroabietic acid ^‡^	
	citramalic acid	X
	4-hydroxybenzoic acid	X
	3,4-dihydroxy benzoic acid	X
	2-deocyerythritol	X
	1-deoxyerythritol	X
high signal intensity	D-sorbitol	X
	Glycerol	X
	p-cresol	X
	stearic acid	X
	glucose ^¶^	
	sulfuric acid ^†^	
	phosphate ^#^	

*^†^Sulfuric acid was not selected because it is not organic, and is an expected product of acidification of sulfate containing medium. ^‡^This metabolite was not selected because the prohibitive cost and low quantities available commercially made it unsuitable for further testing. ^§^Fructose was not selected because previous results indicated that the presence of fructose did not result in improved solubilization (Brisson et al., 2016). ^¶^Glucose was not selected because it was provided in the medium as a substrate, and is expected to be higher for growth on monazite due to the reduced growth rate. ^#^Phosphate was not selected because it is the subject of study, and was provided in the medium as KH_2_PO_4_ in one of the growth conditions.*

#### Metabolites Released at Higher Concentrations Without Soluble Phosphate

Direct comparison of metabolite levels for the two growth conditions identified metabolites with higher concentrations for the monazite-only growth condition (Figure 3). This analysis identified 15 and 13 metabolites for the 2 and 4 day time points, respectively. Three metabolites were identified for both time points. However, none of these three had identification beyond BinBase ID numbers (20282, 2044, and 1681). No metabolites were identified from the analysis at the 6 day time point because differences in concentration were not found to be statistically significant, likely due to the high variability at this time point.

Of the eleven metabolites identified by name (eight for 2 days and three for 4 days) seven (ribose, ribitol, nicotinic acid, isothreonic acid, gluconic acid, histidine, and citric acid) were selected for further study of their leaching abilities using laboratory prepared solutions of these metabolites to leach abiotically. Sulfuric acid was not considered because the focus of this analysis was organic metabolites, and because the acidification of the medium, which contained sulfate, produces sulfuric acid. Glucose was not considered because it was the provided substrate rather than a metabolite and its higher concentration in the monazite-only condition at 4 days was already shown (Figure 1D). Fructose was also rejected because our previous study of monazite bioleaching with this organism tested fructose as a carbon source found that it did not have any benefits over glucose in REE solubilization (Brisson et al., 2016). Ribonic acid was not used in the further experiment because of the prohibitive cost and low quantities that could be purchased commercially. This not only made it unsuitable for further testing, but also makes it a less desirable target for a potential leaching technology.

#### Metabolites With Concentrations That Correlated With REE Concentrations

Fifteen metabolites, eight of which were identified by chemical name, were found to have positive correlations with REE concentrations (Figure 4). Six of these (galactinol, citramalic acid, 4-hydroxybenzoic acid, 3,4-dihydroxybenzoic acid, 1-deoxyerythritol, 2-deoxyerythritol) were selected for further leaching studies, while the other two (erythronic acid and dehydroabietic acid) were rejected for the same reasons at ribonic acid above.

#### High Signal Intensity Metabolites

Seven named metabolites had high overall signal intensities (average signal intensity >105 for at least one condition and time point). Four of these compounds (sorbitol, glycerol, p-cresol, and stearic acid) were selected for further study, while three (glucose, sulfuric acid, and phosphate), were rejected for previously stated reasons.

### Abiotic Leaching Effectiveness of Identified Metabolites

**FIGURE 5:**
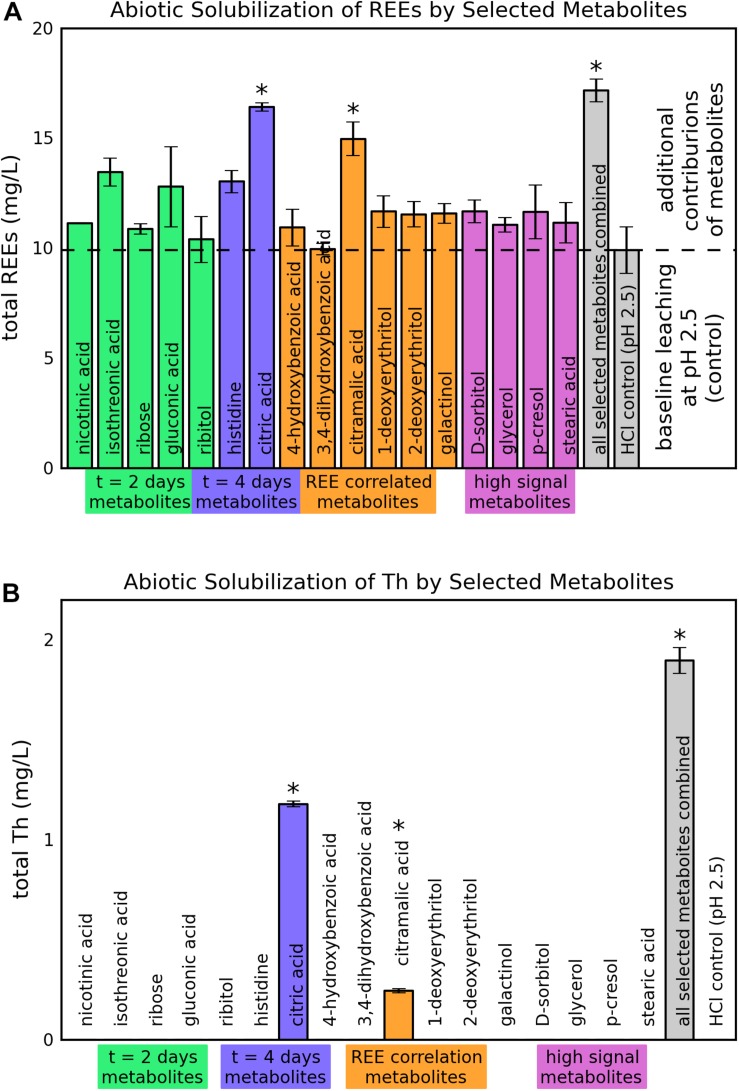
Abiotic solubilization of REEs and Th from monazite by selected metabolites. Heights and error bars indicate means and standard deviations for three replicates. The ^∗^ symbol indicates a statistically significant increase in leaching compared to the control based on an adjusted *p*-value < 0.05. **(A)** REE solubilization. **(B)** Th solubilization.

Of the 17 tested metabolites, two (citric acid and citramalic acid) showed statistically significant improvements in REE solubilization greater than the pH 2.5 HCl control, indicating additional leaching contributions beyond what was expected for pH effects (*p* = 0.008 and 0.04 after Šidák correction for citric and citramalic acid respectively) (Figure 5A). Leaching with a combination of all selected metabolites did not improve solubilization significantly beyond the combined effects of individual metabolites, with increases of only approximately 6.5 and 5.1 mg/L above controls (9.9 mg/L for pH = 2.5 control) for citric and citramalic acids, respectively, and did not approach the REE concentrations achieved by direct bioleaching (42 ± 15 mg/L, see Figure 1A). With regard to Th release during bioleaching, only citric acid, citramalic acid, and the combination of all selected metabolites resulted in detectable levels of Th release, with citric acid releasing significantly more Th than citramalic acid (1.18 ± 0.01 mg/L as opposed to 0.25 ± 0.01 mg/L) (Figure 5B).

## Discussion

This study investigated exuded metabolites as potential contributors to bioleaching of REEs from monazite by a *Paecilomyces* fungus. A previous study found that spent bioleaching medium from this fungus could effectively leach REEs from monazite, indicating that exuded compounds contribute to bioleaching (Brisson et al., 2016). However, the organic acids detected and tested in that study did not account for the leaching capacity of the spent medium. In the study presented here, we employed a metabolomics analysis to identify a larger set of exuded compounds of potential bioleaching interest, and tested their leaching capabilities.

This study focused on the impacts of soluble phosphate availability on the exometabolome of this bioleaching fungus during bioleaching. This comparison was chosen because the fungus may regulate the exudation of bioleaching relevant compounds in response to the need to acquire otherwise unavailable phosphate. Thus, only the soluble phosphate availability was varied, while the presence and amount of monazite was kept constant in order to exclude confounding impacts of the presence or absence of the monazite itself and focus on phosphate availability. Monazite may have other impacts on the fungal exometabolome, potentially in response to the REEs themselves, the radioactive element Th, or the physical presence of the ground sand particles. While these effects could be interesting, they were outside the scope and focus of this study.

The absence of soluble phosphate, and reliance on monazite as the sole phosphate source, resulted in a lag in fungal growth, but was necessary for effective leaching of REEs. By the end of the bioleaching experiment, our data indicate that phosphate was not the limiting factor for growth. This is supported by the comparable glucose, pH, and biomass levels achieved under both growth conditions by the sixth day of bioleaching (Figure 1). REE and Th solubilization were much higher when monazite was the only phosphate source. This is consistent both with forcing the organisms to solubilize phosphate from monazite for growth and with possible re-precipitation of REE-PO4 in the medium that contains K_2_HPO_4_ at a relatively high phosphate content. The solubilities of REE phosphates are known to be extremely low (approximately 10–13 M) (Firsching and Brune, 1991).

The depletion of glucose by the end of the experiment under both growth conditions may suggest a glucose growth limitation. However, in a previous study with the same organism (Brisson et al., 2016), increasing the glucose concentration to 100 g/L did not improve bioleaching performance. Nitrogen availability is another possible growth limiting factor. Some estimates of the nitrogen content of fungal mycelia range from 0.2 to 9% of dry weight (Lahoz et al., 1966; Dawson et al., 1989; Watkinson et al., 2006). Assuming a typical value of 5% nitrogen content, the 0.66 g/L of (NH_4_)_2_SO_4_ (i.e., 0.14 g/L N) provided in AMS medium would correspond to the production of approximately 2.8 g/L of dry biomass. This is comparable to the biomass produced under both growth conditions in this study, suggesting that nitrogen availability may be limiting biomass production to this level. Scervino et al. (2011) found nitrogen limitation to enhance phosphate mineral solubilization by *Penicillium purpurogenum*, another phosphate solubilizing fungus, indicating that nitrogen limitation of growth may be desirable for bioleaching performance. Nitrogen limitation has also been found to enhance citric acid production in some fungi (Cunningham and Kuiack, 1992; Papagianni, 2007).

Hierarchical clustering (Müllner, 2011) of metabolites based on their concentration profiles identified groups of metabolites with similar responses to the two growth conditions and progression over the course of fungal growth. As the fungus progressed from exponential growth (day two for growth with K_2_HPO_4_, days two and four for monazite only), through a transition (day four for growth with K_2_HPO_4_, day six for monazite only), and into stationary phase (day six for growth with K_2_HPO_4_) (see Figure 1F), there were distinct groups of metabolites detected at each stage (Figure 2). Metabolites that clustered together and were detected at higher concentrations in the earlier stages include several disaccharides (cellobiose, lactulose, and β-gentiobiose) and alcohols (lactitol, lyxitol, and glycerol) (Figure 2, yellow highlights).

Most of the components of the TCA cycle (Figure 2, green highlights) clustered together, with concentrations peaking near the transition from exponential growth to stationary phase (day four with K_2_HPO_4_ and day six for monazite only). This overall trend is consistent with the earlier depletion of glucose when K_2_HPO_4_ was provided (Figure 1G) since glucose, through glycolysis and the TCA cycle, feeds into the production of these metabolites (Madigan et al., 2008). Once the glucose is depleted, these TCA cycle components are consumed and not replenished, resulting in the reduced concentrations by day six when K_2_HPO_4_ is provided (Figure 2). The importance of maintaining high sugar concentrations for the production and excretion of citric acid by *Aspergillus niger*, a commercially important production process, have been well documented (Magnuson and Lasure, 2004; Papagianni, 2007).

Long chain fatty acids (longer than eight carbons) (Figure 2, purple highlights) also clustered together, with concentrations peaking on day six when K_2_HPO_4_ was provided, and remaining at much lower levels when monazite was the only phosphate source. Long chain fatty acids observed were azelaic, capric, lauric, oleic, palmitic, pelargonic, and stearic acids. This increase in long chain fatty acid production corresponds with the depletion of glucose and the leveling off in biomass production (Figures 1D,F), and may be related to a transition from exponential growth to stationary phase. Long chain fatty acids and their derivatives have been associated with changes in fungal physiology and morphology, and specifically with the transition from growth to spore formation (Mysyakina and Feofilova, 2011).

Analysis of individual metabolite levels identified several metabolites potentially correlated with bioleaching, and further experiments with 17 of these metabolites showed that citric and citramalic acids contribute significantly to monazite leaching. A combination of all 17 metabolites together did not increase solubilization beyond the expected contributions of the individual metabolites, indicating that there were no synergistic effects of the combination of these metabolites.

Previous studies have shown that citric acid was more effective that oxalic, phthalic, salicylic, gluconic, itaconic, succinic, and acetic acids at leaching REEs from monazite (Goyne et al., 2010; Brisson et al., 2016). Although the effect of citric acid appears to be somewhat larger here than that reported in our previous study of monazite leaching (6.5 mg/L here as opposed to 3 mg/L in our previous study) (Brisson et al., 2016), the experimental protocols were quite different (see Materials and methods). This experiment supports the overall result from that study, indicating that citric acid provides some additional REE solubilization, although not sufficient improvements to account for the majority of the bioleaching effectiveness.

To our knowledge, REE solubilization by citramalic acid has not been previously reported. One study found that phosphate stress induced increased levels if citramailc acid exudation from the roots of sugar beet plants (Khorassani et al., 2011). That study also showed that citramalic acid solubilized phosphate from low phosphate soils amended with monocalcium phosphate dihydrate (Ca(H_2_PO_4_)2⋅H_2_O) (Khorassani et al., 2011).

Notably, the same metabolites that contributed to additional REE solubilization also contributed to Th solubilization. However, citric acid leached significantly more Th than citramalic acid, indicating that citramalic acid may have more desirable leaching characteristics for preferential leaching of REEs. Th, which is radioactive, commonly co-occurs in monazite with REEs-phosphates, and can present concerns for downstream processing and waste disposal in monazite extraction (Gupta and Krishnamurthy, 1992).

The ability of citric and citramalic acids to leach REEs, as well as their differential levels of Th leaching, make them interesting targets for development of an industrial bioleaching process. Fungi are known to produce a wide array of organic acids, and are used industrially for the production of citric, gluconic, itaconic, kojic, and oxalic acids (Liaud et al., 2014; Yang et al., 2017; Hossain et al., 2019). Of the 87 metabolites identified in this study, 37 were organic acids (Supplementary Table 1), and eight of the 17 metabolites selected for abiotic leaching tests were organic acids. Only two metabolites, citric and citramalic acid, significantly increased leaching. This result indicates that the particular organic acids, rather that organic acids in general, are important for effective leaching. As another example, oxalic acid, which is also produced by fungi, forms insoluble complexes with REEs and causes them to precipitate out of solution (Brisson et al., 2016). The differences in Th solubilization between citric and citramalic acid further emphasizes the importance of the particular acids present. A recent study describing a citramalic acid biosynthesis pathway in *A. niger* showed that overexpression of two genes, *cimA* and *mfsB*, reduced citric acid production and increased citramalic acid production (Hossain et al., 2019). This type of control could be useful for developing a process to preferentially recover REEs without the release of Th.

This study investigated 17 metabolites that were potentially associated with bioleaching and could be identified and tested for their leaching abilities. However, a large proportion of detected metabolites (123 out of 210) could not be identified, and 21 of these unidentified metabolites were either correlated with REE concentration or were exuded at higher concentrations when soluble phosphate was not provided. Some of these unknown compounds may also contribute to bioleaching activity, but without identifications their contributions could not be investigated in this study. However, as mass spectrometry databases continue to expand, it may be possible to identify these compounds in the future based on their retention indices and mass spectra (Supplementary Table 1).

In addition to the metabolites detected by the methods used in this study, other products exuded by the fungus, such as siderophores or extracellular enzymes, may also contribute to bioleaching. Although generally studied in the context of iron complexation, siderophores have also been shown to form complexes with REEs, and could also contribute to bioleaching (Bau et al., 2013; Osman et al., 2019). Siderophore production and protein excretion by the strain used in this study have not been characterized. However, the related species *Paecilomyces variotii* has been shown to produce multiple siderophores (Vala et al., 2000). *P. variotii* is also known to produce a variety of extracellular enzymes, including phytases, which breaks down phytic acid by removing phosphate groups (de Laguna et al., 2015).

This work has advanced our understanding of the monazite bioleaching process by a *Paecilomyces* fungus. The metabolomic analysis identified the impacts of soluble vs. insoluble phosphate availability on the dynamics of metabolite exudation. Based on that analysis, we identified and tested a set of metabolites of potential bioleaching importance. We confirmed the contribution of citric acid and identified an additional contribution by citramalic acid to the bioleaching process. Further, we showed that citramalic acid exhibits preferable leaching properties to those of citric acid due to the reduced Th release.

## Data Availability Statement

The GC-MS metabolomics data have been deposited in the Global Natural Product Social Molecular Networking (GNPS) data repository with the MassIVE (Mass Spectrometry Interactive Virtual Environment) accession number MSV000084613.

## Author Contributions

All authors designed and planned the experiments, contributed to the discussion of the results, and wrote the manuscript. VB conducted the experiments and analyzed the data.

## Conflict of Interest

The authors declare that the research was conducted in the absence of any commercial or financial relationships that could be construed as a potential conflict of interest. The handling Editor declared a shared affiliation, though no other collaboration, with the author LA-C at the time of review.
