# Supplementary material for: Metabolomic Analysis Reveals Contributions of Citric and Citramalic Acids to Rare Earth Bioleaching by a Paecilomyces Fungus
Source: Front Microbiol. 2020 Jan 14;10:3008. doi: 10.3389/fmicb.2019.03008 (PMC6971059; doi:10.3389/fmicb.2019.03008)
Supplement: Supplementary file 1 [file Image_1.pdf]

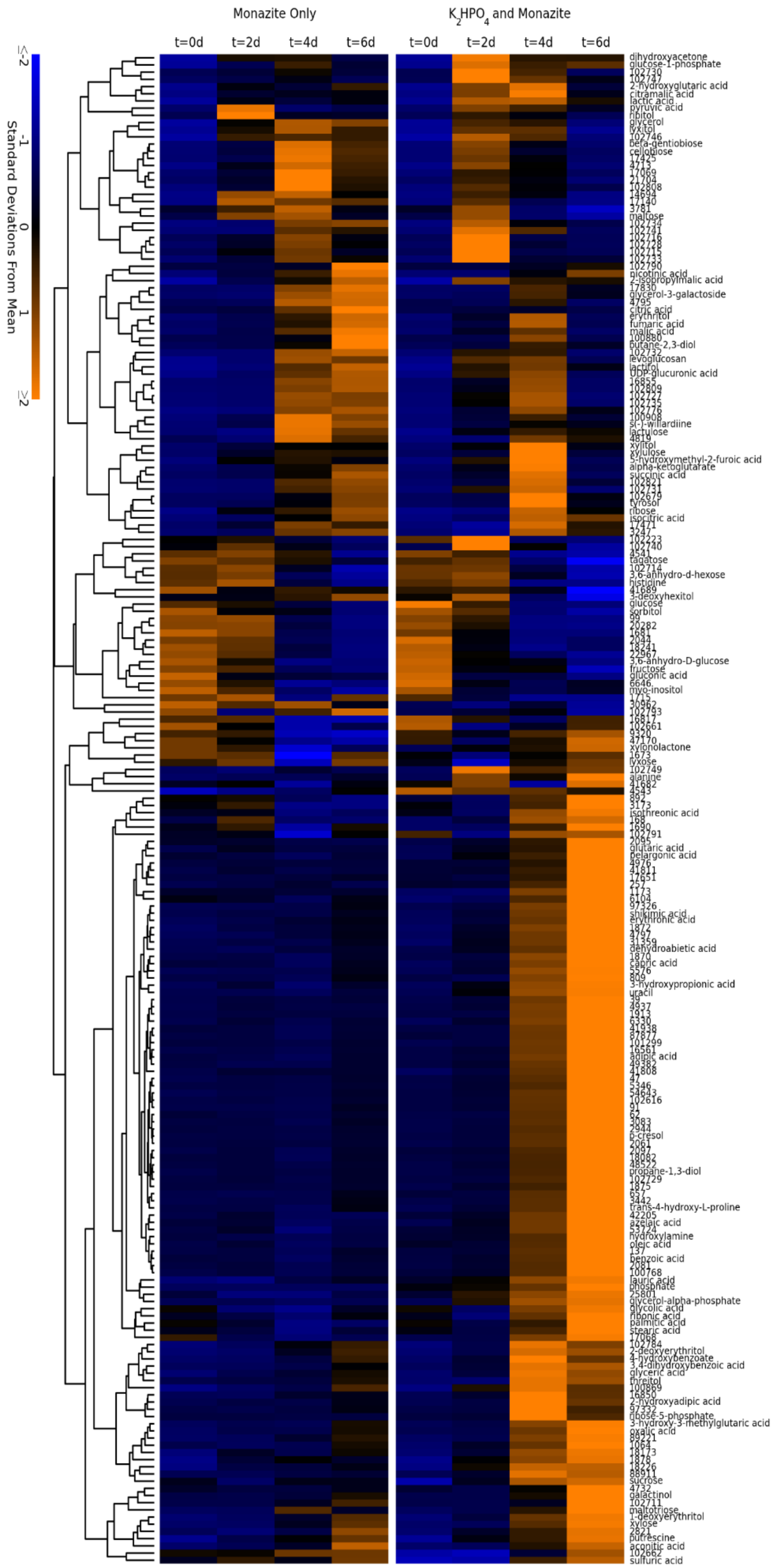

Supplementary Figure 1. Concentration profiles of all detected metabolites, including those identified only by BinBase IDs. Heatmap shows average levels of metabolites detected during monazite bioleaching for each growth condition and time point. Rows represent different conditions and time points. Columns represent different metabolites. Metabolites are ordered based on hierarchical clustering, with the clustering dendrogram displayed at the bottom of the heatmap and metabolite names to the right. Heatmap colors indicate standard deviations below (blue) and above (orange) the overall mean level for each metabolite.
